# Direct Pulp-Capping with Calcium Enriched Mixture in Primary Molar Teeth: A Randomized Clinical Trial

**Published:** 2010-02-20

**Authors:** Masoud Fallahinejad Ghajari, Tahereh Asgharian Jeddi, Sonay Iri, Saeed Asgary

**Affiliations:** 1. Department of Pediatric Dentistry, Dental Research Center/Dental School, Shahid Beheshti University of Medical Sciences, Tehran, Iran.; 2. General Dentist, Tehran, Iran.; 3. Department of Endodontics, Iranian Center for Endodontic Research, Dental Research Center/Dental School, Shahid Beheshti University of Medical Sciences, Tehran, Iran.

**Keywords:** Calcium Enriched Mixture, CEM Cement, Direct Pulp Capping, MTA, NEC, Primary Molar, Vital Pulp Therapy

## Abstract

**INTRODUCTION:**

The aim of this trial was to compare clinical and radiographic success rates of direct pulp capping (DPC) using a novel biomaterial called Calcium Enriched Mixture (CEM) cement versus Mineral Trioxide Aggregate (MTA) in primary molar teeth.

**MATERIALS AND METHODS:**

In this randomized clinical trial 42 deciduous molars in 21 patients who had at least two teeth requiring DPC, were treated. The enrolled patients were between 5-8 years. The molar teeth were randomly divided into two experimental groups. Patients and operators were blinded. The teeth were anaesthetized, caries were removed and after pinpoint exposure of dental pulp, haemostasis was achieved. The exposure points were capped with MTA or CEM cement. All teeth were restored with amalgam. Patients were recalled for the 6-month follow up. Statistical analysis was carried out using McNemar test.

**RESULTS:**

Thirty-eight teeth were available for follow up (19 in each group). The radiographic evaluations did not show failure in experimental groups; however, in clinical examinations one sinus tract was found in CEM cement group. Clinical success rates in CEM cement and MTA groups were 94.8% and 100%, respectively. Dentinal bridge formation was not observed in the two experimental groups.

**CONCLUSION:**

There is no significant difference between treatment outcomes of direct pulp capping with either CEM cement or MTA; therefore, both biomaterials can be used successfully for DPC in primary molar teeth.

## INTRODUCTION

In spite of the recent progress in preventive dentistry and the increasing importance of tooth preservation, primary teeth are still lost early leading to malocclusions and aesthetic disorders [1][2]. Therefore, maintaining teeth and surrounding tissues is essential [3]. Vital pulp therapies (VPT) are common treatments in pedodontics which help to preserve pulp vitality; direct pulp capping (DPC) is less invasive than pulpotomy or pulpectomy [4]. However, the use of DPC is more limited in primary teeth compared with permanent teeth [5]. Numerous materials have been used for DPC including calcium hydroxide (CH), ZOE cement, formocresol, polycarboxylate cements, inert materials, adhesives, enamel matrix derivative (EMD), beta-tricalcium phosphate and mineral trioxide aggregate [4][5][6][7].

Calcium hydroxide was introduced by Herman in 1930 as a pulp capping agent [8]. The antibacterial effect of CH is a desirable property for pulp capping materials [9]. Different studies have reported successful results using CH for DPC in primary teeth [10][11]. However, the slow stimulation of dentine bridge formation and long-term solubility and degradability of CH, causes microleakage as well as tunnel defects, leading to the induction of internal resorption and consequent tooth loss [12][13][14].

Mineral trioxide aggregate has been introduced as a superior material for dental pulp capping [15]. MTA is an endodontic biomaterial with superior sealing ability and biocompatibility. This material is less cytotoxic than other conventional materials currently used in VPT [15]. It can stimulate the repair of the dental pulp, PDL and surrounding bone [16]; however it is an expensive material with long setting time and potential discoloration [17].

Calcium enriched mixture (CEM) cement, a recently introduced biomaterial, has good biocompatibility [12][18], ability to induce hard tissue formation [14], including hydroxyapatite [22][23], and it can resist microbial re-entrance [12]. CEM cement has appropriate sealing ability. Sets in aqueous environments [19], remarkable antibacterial activity [21] and quick setting time (<1 hour) [19].

This is the first in vivo study which evaluates this novel material for DPC of primary molar teeth. The aim of this randomized controlled, quadruple-blind and prospective trial was to evaluate the clinical and radiographic success rates of CEM cement compared with MTA (gold standard) in DPC of primary molars of 5-8 year old children.

## MATERIALS AND METHODS

The study protocol was approved by Iranian Center for Endodontic Research (ICER) and the Ethics Committee of Dental Research Center of Shahid Beheshti Medical University, Tehran, Iran. The trial was conducted in compliance with the ethical principles of The Helsinki Declaration.

In this quadruple-blind randomized clinical trial, 21 patients (5-8 years old) met the inclusion criteria. Procedure was thoroughly explained to the parents, and a written informed consent taken.

Teeth had to have appropriate clinical and radiographic sign/symptoms: 1) carious primary tooth with vital pulp; 2) radiographic observation of presence of at least two-thirds root length. Inclusion criteria for performing DPC and coronal restoration were as follows: 1) patients with at least two carious primary molars with vital pulp, 2) availability for 6-months follow up; and 3) have no systemic disease or special drug use; as well as 4) provided and agreed to the written informed consent. Patients with a history of spontaneous pain, tooth tender to percussion, absence of underlying permanent teeth, internal/external root resorption, apical/furcal lesions, sinus tract, periodontal pocket >3mm, physiologic or pathologic luxation, and/or presence of abscess were excluded from the study.

Teeth were anesthetized using lidocaine 2% with epinephrine 1/80000 (Daroupakhsh, Tehran, Iran) and then isolated with cotton rolls and suction. Enamel caries were removed using diamond fissure bur 008 with high speed handpiece and copious water supply. Dentinal caries were removed using carbide round bur. In cases where pulp exposures was <1 mm and bleeding arrested in <3 min, DPC were performed. The teeth with exposures >1 mm were excluded and subsequently pulpotomized. The cavities of DPC cases were irrigated with sterile normal saline solution and then dried with sterile cotton pellet. A wet cotton pellet was placed with a slight force in the cavity. After hemostasis, MTA (ProRoot, Tooth colored; Dentsply, Tulsa, OK, USA) and CEM cement (Tehran, Iran) were prepared into creamy mixtures by the blind practitioners and then placed over the exposures. A dry cotton pellet was pressed slightly over the cements for further adaptation of the capping material with dental pulp orifices.

All teeth were immediately restored with amalgam. The teeth were evaluated at 6 month followed up. The outcome of clinical and radiographic success or failure was determined by the criteria outlined below. Note that the presence of one or more of these clinical and/or radiographic symptom/signs determined the case as a failure.

1) Symptoms such as pain, swelling, tenderness to pressure, and signs such as presence of sinus tract, swelling and tenderness to percussion were evaluated as the clinical criteria for failure and;

2) internal and/or external root resorption, interradicular radiolucencies, and periapical lesions were assessed as the radiographic criteria for failure.

One blinded pedodontist examined the patients clinically and two blinded radiologists inter-preted the radiographs. Data were analyzed using SPSS software version 13 (SPSS Inc., Chicago, IL, USA). McNemar test was also used for comparing the frequency of success and failure of treatments after 6-month follow up.

## RESULTS

Forty two primary second molar teeth in 21 patients were treated with DPC. The mean age of patients was 6.9 ± 0.7 (5 boys with the mean age of 6.6 ± 0.2 years and 16 girls with the age range of 7 ± 0.6 years). Nineteen maxillary teeth (45.23%) and 23 mandibular teeth (54.77%) were treated. All patients except two were assessed at 6 month follow up appointment. Therefore, a total of 19 teeth in the MTA group and 19 teeth in CEM group were evaluated.

Pain, swelling, tenderness to percussion, or pathologic luxation was not observed in the studied teeth. One tooth treated with CEM cement showed a sinus tract. Analysis with intention to treat protocol revealed 18/21 and 19/21 successful result for CEM cement and MTA, respectively, which was not statistically significant (P=0.721). No radiographic failure was observed in both groups at 6 months.

## DISCUSSION

Previous studies demonstrated that direct pulp capping may have poor prognosis in primary teeth. They are likely to suffer from internal resorption, probable pulp calcification, necrosis and injuries to surrounding tissues [3][11]. Fuks stated that undifferentiated mesenchymal cells in the primary pulp differentiated to odontoclasts, leading to internal resorption [3]. However, DPC has the advantage of being a conservative vital pulp therapy [6][9] reducing the need for more invasive treatments [4]. At the 6 months follow-up appointment, all the cases (except one) remained vital, with no sign or symptoms, suggesting reasonable success for DPC in primary teeth.

Numerous materials have been used for DPC in primary teeth; among which calcium hydroxide and MTA are more common [24]. Several studies have shown that MTA application for DPC of human deciduous teeth with mechanical pulp exposure causes thicker dentin bridge formation and less inflammation compared to CH [25][26]. Dental pulp capping with MTA has shown very favorable results [9][25][26]; therefore this material was considered as the gold standard in this trial.

Our trial showed no clinical signs including pain, swelling, pathologic luxation, and tenderness to percussion was observed. Only one tooth with fistula was detected in CEM cement group. The success rate of DPC treatment using MTA has been reported 100% after 1-24 months follow up in a recent study [26], concurring with our study. Another study which analyzed the dental pulp response to pulpotomy and DPC using MTA, showed positive clinical results after 5-months follow up [9]. These clinical successes can be due to thorough removal of caries and bacteria by the operator, and the hermetic seal [20], antibacterial effect [21], low cytotoxicity [18], biocompatibility and hard tissue formation [12][14] of the two biomaterials.

Interradicular lesions are more common in primary teeth compared with periapical lesions [27]. No radiographic sign indicating treatment failure was observed in this study; therefore the radiographic success rate for two experimental groups was 100%. This is in accordance with other studies [9][10][11][12][13][14][15][16][17][18][19][20][21][22][23][24][25][26].

DPC and amalgam restoration were performed in one-visit as exposures were small pinpoint ones and to increase time efficiency. Good adaptation between pulp capping materials and cavity walls can be achieved by using wet cotton pellets on the DPC material, and condensing the amalgam using large size pear shape condenser with gentle pressure.

Tuna and Olmez claimed that the successful results of CH and MTA as direct pulp capping materials is due to the subsequent placement of ZOE as the baseliner, which provides a long-term hermetic seal [26]. Although using baseliners over the used pulp capping biomaterials (MTA and CEM cement) can be considered; this study demonstrated excellent results with immediate amalgam restoration without placement of baseliners.

## CONCLUSION

This study indicated that CEM cement can be used as an appropriate biomaterial for DPC of deciduous teeth. Similar investigations on this material with larger sample size and protracted follow ups are suggested.
